# Rare cavitary lymphoepithelioma-like carcinoma of lung: clinical experience and literature review

**DOI:** 10.1186/s12890-023-02529-x

**Published:** 2023-07-05

**Authors:** Guiqin Chen, Qiane Yu, Haifeng Ran, XuHong Li, Tijiang Zhang

**Affiliations:** Department of Radiology, Medical Imaging Center of Guizhou Province, the Affiliated Hospital of Zunyi Medical University, Zunyi, 563000 China

**Keywords:** Lymphoepithelioma-like carcinoma, Lung carcinoma, Cavity, Computed tomography, Positron emission computer tomography

## Abstract

**Background:**

Lymphoepithelioma-like carcinoma of the lung is a rare primary malignancy of the lung, accounting for only 0.9% of primary malignancies of the lung. Those associated with cavities are even rarer, with fewer than five cases reported in the English literature. Concurrently, the imaging findings of tumors are usually non-specific, resulting in insufficient understanding of the disease by clinicians, thus leading to misdiagnosis and delayed treatment.

**Case presentation:**

A 42-year-old female presented with a right lower lung mass with cavities. First identified on chest computed tomography (CT) in 2021, the mass persisted for 1 year and subsequently enlarged on chemotherapy and routine follow-up CT. Right lower lobectomy was then performed. Postoperative pathology confirmed primary pulmonary lymphoepithelioma-like carcinoma. After 10 months of follow-up, the patient was still alive and no recurrence was observed.

**Conclusions:**

This article aims to describe a rare case of cavitary lymphoepithelioma-like carcinoma of the lung and review it clinical and imaging characteristics reported in previous cases, which will be helpful for clinicians and imaging physicians in diagnosing this disease.

## Background

Lymphoepithelioma-like carcinoma (LELC) is a non-keratinizing carcinoma that often occurs in the nasopharynx and foregut derived organs, such as the stomach, thymus, and liver [1]. In the literature, 56.22% of LELCs occurred in the nasopharynx, followed by 21.32% in the non-nasopharyngeal head and neck, and 7.83% in the respiratory system [2]. The incidence of primary pulmonary LELC is rare, making up only 0.9% of all primary lung cancers [3]. Women and non-smokers are generally affected by pulmonary LELC, which has a strong association with Epstein-Barr virus [4]. LELC is nonspecific, usually resembling bronchial carcinoma, and present as a solid soft tissue mass in the lung [5]. In rare cases the tumor unexpectedly shows a cavity. It is difficult to distinguish pulmonary LELC from other lung cancers on imaging. Here, we report a rare case of cavitary lymphoepithelioma-like carcinoma of the lung, which has few been reported before, and review the previous clinical and imaging characteristics associated with pulmonary LELC to provide radiologists with a broader idea in the diagnosis and differential diagnosis of pulmonary tumors.

## Case presentation

**Fig. 1:**
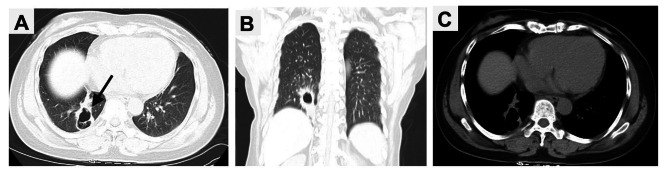
A 42-year-old female patient underwent routine CT examination on January 06, 2022, and found a round-like cavity in the right lung with irregular inner wall, and its size was about 59 mm×31 mm (black arrow). **(A)** Axial lung window;**(B)** Coronal lung window; **(C)** Axial soft tissue window

**Fig. 2:**
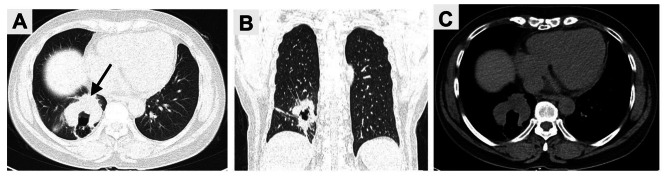
Follow-up CT examination on May 11, 2022 showed that the mass was obviously enlarged and the solid part increased, and its size was about 51 mm×61 mm (black arrow). **(A)** Axial lung window; **(B)** Coronal lung window; **(C)** Axial soft tissue window

**Fig. 3:**
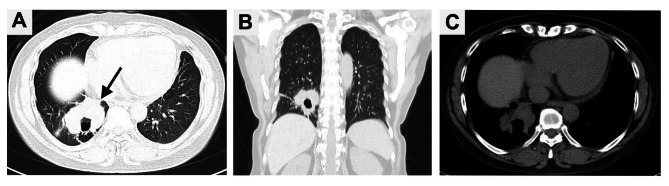
Follow-up CT examination on June 7, 2022 showed that the mass continued to enlarge and the solid part increased, and its size was about 58 mm×63 mm (black arrow). **(A)** Axial lung window; **(B)** Coronal lung window; **(C)** Axial soft tissue window

**Fig. 4:**
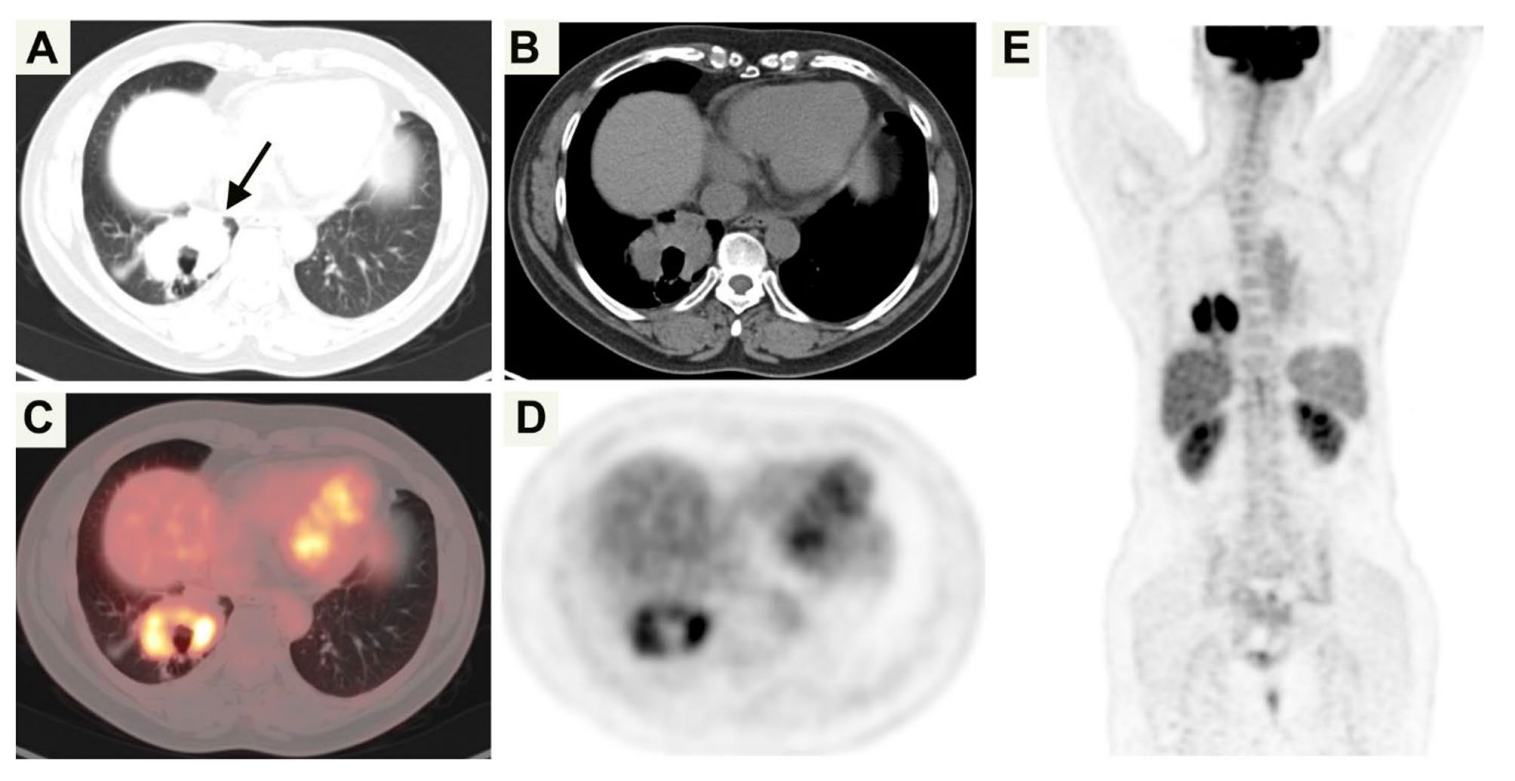
^18^ F-FDG PET/CT images. Right lung lesion showed an irregular mass, central cavity and annular marked FDG uptake with maximum standardized uptake value (SUVmax) of 20.7 (black arrow); **(A)** Axial lung window; **(B)** Axial soft tissue window ; **(C)** PET/CT fusion image;**(D)** PET ; **(E)** Maximum intensity projection image of whole body

**Fig. 5:**
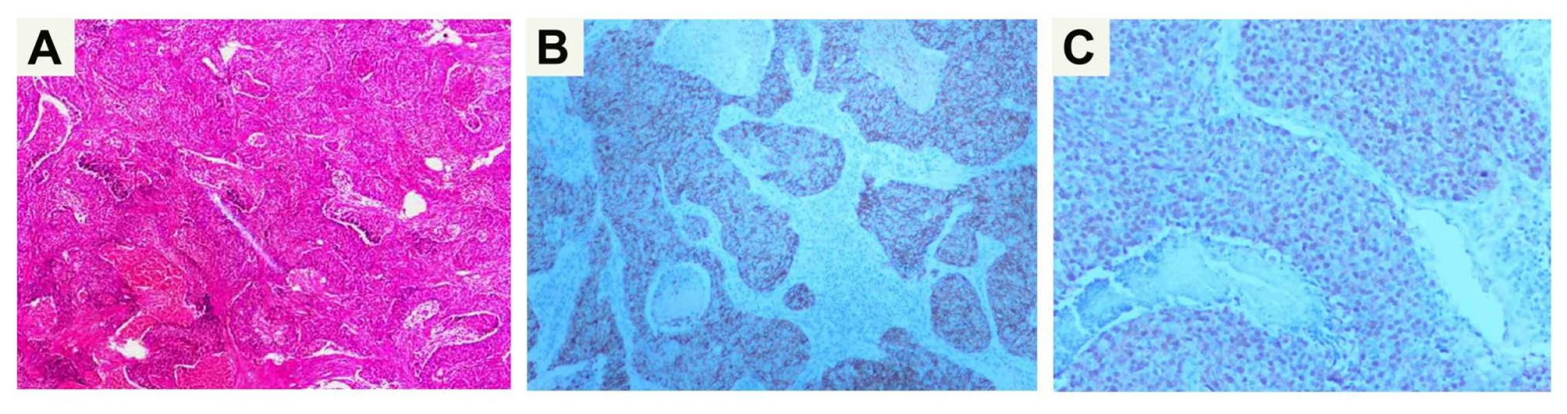
**(A)** Hematoxylin and eosin(HE) staining showing the tumor cells were mixed with lymphocytes (×50); **(B)** The expression of CK5/6 in the cancer cells was positive by immunohistochemical staining (×50); **(C)** The molecular pathological result was EBER/ISH (+) (×10)

A 42-year-old woman presented to our hospital with cough and expectoration for more than one year. There were no symptoms of fever, chest pain, nausea and vomiting. Upon physical examination, the bilateral thorax was symmetrical, and the respiratory sounds of both lungs were clear. The sound was clear after percussion, and no dry-wet rales were heard. There was no abnormality in the laboratory examination. The chest CT scan showed an irregular mass (59 mm×31 mm) in the right lower lobe, with irregular cavities, uneven inner cavity walls, and mural nodules (Fig. 1). Because it was difficult to diagnose only by imaging and laboratory examination, the patient underwent lung biopsy. The pathology after biopsy of the right lung showed poorly differentiated carcinoma, combined with immunohistochemistry in line with LELC. According to the nature of the tumor, the patient received 6 rounds of chemotherapy with paclitaxel, sintigerism and carboplatin injection for 5 months. During the follow-up period, chest CT showed that the solid part of the tumor was growing (Figs. 2 and 3). Positron emission tomography-computer tomography (PET-CT) showed a space-occupying lesion with increased metabolism in the lower lobe of the right lung, and a malignant neoplastic lesion was considered (Fig. 4). A video-assisted thoracoscopic right lower lobe tumor resection was performed. Postoperative histopathology showed a large number of tumor cells and plasma cells as seen by hematoxylin and eosin (HE) staining. The tumor cells had scanty cytoplasm, irregular nuclei, large nucleoli, and were dark in color. Mitoses were active and plasma cells were abundant (Fig. 5A and B). Molecular pathology revealed the tumor to be EBER/ISH (+) (Fig. 5C). On the eighth postoperative day, the patient recovered and was discharged without surgery-related complications. After the operation, the patient was followed up via telephone for 10 months. There was no particular discomfort or related complications.

## Discussion and conclusions

**Fig. 6:**
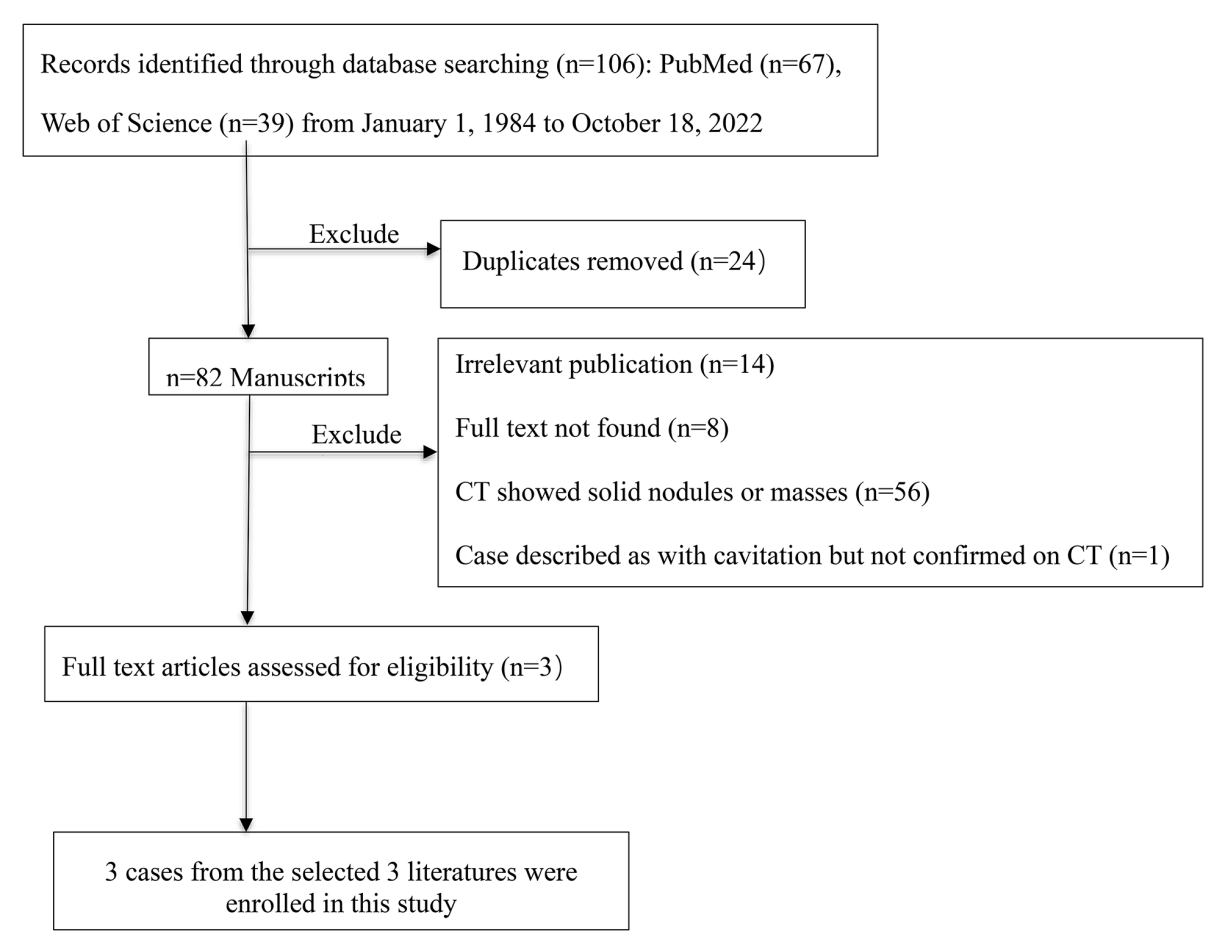
The flow chart of the literature screening process for lymphoepithelioma-like carcinoma of lung

A literature review was performed using relevant articles in English from the PubMed and Web of Science databases. The period of research was set from January 1, 1984 to October 18, 2022, using the following keywords: “lung”, “lungs”, “pulmonary”, “lymphoepithelioma-like carcinoma”, and “lymphoepitheliomatous carcinoma”. These words were used individually “OR” with the Boolean operator “AND”. A total of 82 articles were analyzed from 1984 to 2022. The flow chart of the literature screening process is described in Fig. 6. Finally, three 3 articles involving three cases were included for analysis. For each case, the first author’s name, year of publication, and patient’s age, sex, size, symptoms, tumor location, imaging characteristics, and follow up results were recorded (Table 1). Our results showed that pulmonary LELC had previously appeared in patients between 35 and 76 years of age, and most of these patients presented with nonspecific symptoms such as cough and sputum production. However, only four of them had combined cavities (including ours). Of the four patients, one was male and three were females, The lesions were located in the right lung in three patients, all four patients underwent surgery, and three patients were discharged from the hospital.

**Table 1:** Review of the literature on pulmonary lymphoepithelioma-like carcinoma with cavitary presentation

Case (No.)	References	Sex	Age	Presentation	Tumor size (mm)	Image feature	Operation	Outcome	Follow-up (months)
1	Zeng et al.	female	55	cough and intermittent haemoptysis for 2 years	31 × 25	thin walled cavity with 31 mm× 25 mm located in the right lower lobe	Yes	Discharged	NM
2	Oi et al.	male	69	A detailed examination of an abnormality detected in a routine chest X-ray examination.	17 × 15	Chest CT revealed two round solid masses(17 mm and 15 mm) with a circumscribed border arising from a thin-walled cavity located in the left S9 area and in the vis-ceral pleura of the left lower lobe, respectively, and one enlarged lymph node (20 mm) in the left hilum	Yes	death	6
3	Hsieh et al.	female	53	had experienced cough and hemoptysis for 3 weeks	50	a 50 mm cystic lesion over the right upper lobe	Yes	Discharged	18

F, female; M, male; CT, computed tomography; NM, not mentioned

We report a 42-year-old female patient who presented with a right lower lung mass with cavities on CT, having thick and irregular cavity walls and small mural nodules. Combined with the clinical presentation and chest CT findings, a malignant lung tumor was highly suspected by clinicians. Some studies have reported [6] that pulmonary cavitary lesions are commonly found in pulmonary tuberculosis and lung squamous cell carcinoma. Tuberculosis or aspergillosis cavities often have smooth thin walls, while squamous cell carcinoma of the lung usually has other malignant characteristics including irregular margins and cavities with thick wall. In our case, the lesion presented as a lung mass with irregular thick-walled cavities and mural nodules, strongly suggesting squamous cell carcinoma. However, surprisingly, the final pathological findings revealed the tumor to be LELC rather than squamous cell carcinoma of the lung.

LELC is a rare lung cancer that accounts for only 0.9% of all primary lung cancers [7]. The World Health Organization (WHO) classifies it as a subtype of large cell carcinoma [8]. Bégin et al. first reported the disease in 1987 [4]. Smoking does not seem to have a significant impact on primary pulmonary LELC, unlike non-small cell lung cancer [9]. Primary pulmonary LELC has few specific clinical manifestations at the time of diagnosis, and larger tumors may presents with a variety of clinical signs and symptoms, such as cough, sputum production, hemoptysis, chest pain, and weight loss [10].

CT remains an important examination method for the diagnosis and differential diagnosis of pulmonary LELC [11]. These tumors are mainly located in the lower lobes of both lungs, close to the mediastinum and the pleura, and present as a round, round-like or lobulated solid mass. In the later stage of the disease, the bronchi and large vessels may be invaded, forming a vascular embedding sign, which is rarely accompanied by a cavity [7]. They mainly show high ^18^ F-FDG uptake on PET-CT [9]. Because this disease was combined with cavities, it was difficult to differentiate from common cavitary lesions in the lung, such as peripheral lung cancer, pulmonary tuberculosis and lung abscess. Cavities are found relatively frequently in primary lung cancer, with an incidence of up to 22% on CT [12], with squamous cell carcinoma being the main culprit. Squamous cell carcinoma usually shows eccentric thick-walled cavities with uneven walls, visible burrs, and a pleural indentation sign [13]. Therefore, pulmonary LELC is difficult to distinguish from pulmonary squamous cell carcinoma. Tuberculosis-associated cavities account for about 40% of cases, with most being central cavities with even wall thickness. Sometimes liquid level is observed in the cavity along, with satellite foci [14]. The cavity wall of a lung abscess is generally smooth, and a gas-liquid level or liquid-liquid level can be seen inside [12, 15].

At present, pathological examination is the only method for the diagnosis of LELC [16]. Immunohistochemistry commonly shows positivity for CK, EMA, CK5/6, P40, and p63 in LELC [17]. Treatment is determined based on the cancer stage and comorbidities. Treatment modalities include surgery, chemotherapy, radiotherapy and even targeted therapy. The most effective treatment for early pulmonary LELC is complete resection of the tumor with very good results. In the advanced stage, combination therapy is the best option [18]. Lymphoepithelioid cancer has a better prognosis than other types of lung cancer [19].

Pulmonary LELC has been reported in most cases in the form of solid soft tissue masses in the lung. In rare cases, tumors unexpectedly exhibit cavitary lesions; to our knowledge there are only four cases (including our case), thus easily misleading physicians to diagnose other tumors of the lung or other diseases. Our case demonstrates that LELC can have manifestations other than solid soft tissue masses, but also demonstrates that thick-walled cavities are not limited to common malignant tumors of the lung such as squamous cell carcinoma of the lung.

In conclusion, we report a case of pulmonary LELC in a patient who presented with rare cavitation on chest CT. Although very rare, LELC should be considered in the diagnosis and differential diagnosis of pulmonary cavitary lesions of unknown origin.

## Data Availability

All data generated or analysed during this study are included in this published article.

## Abbreviations

CT: Computed tomography
LELC: Lymphoepithelioma-like carcinoma
PET-CT: Positron emission tomography-computed tomography
HE: Hematoxylin and eosin
